# Supplementary material for: The impacts of climate change on the global range of Culicoides punctatus (Meigen, 1804) with notes on its status in Saudi Arabia
Source: PeerJ. 2025 Feb 13;13:e18916. doi: 10.7717/peerj.18916 (PMC11830369; doi:10.7717/peerj.18916)
Supplement: Supplemental Information 1 — The total 1217 records of Culicoides punctatus. [file peerj-13-18916-s001.pdf]

Sp.	Latitude	Longitude
Culicoides punctatus	58.11	7.93
Culicoides punctatus	58.11	7.93
Culicoides punctatus	58.33	8.24
Culicoides punctatus	58.07	7.98
Culicoides punctatus	61.42	11.1
Culicoides punctatus	58.61326	6.098003
Culicoides punctatus	58.55675	5.806088
Culicoides punctatus	58.61794	5.706579
Culicoides punctatus	58.55675	5.806088
Culicoides punctatus	59.25599	5.223407
Culicoides punctatus	59.51861	5.566979
Culicoides punctatus	59.25599	5.223407
Culicoides punctatus	58.55675	5.806088
Culicoides punctatus	58.61794	5.706579
Culicoides punctatus	58.61794	5.706579
Culicoides punctatus	59.51861	5.566979
Culicoides punctatus	59.25599	5.223407
Culicoides punctatus	58.61794	5.706579
Culicoides punctatus	58.61794	5.706579
Culicoides punctatus	59.25599	5.223407
Culicoides punctatus	58.55675	5.806088
Culicoides punctatus	59.67705	11.28236
Culicoides punctatus	58.55675	5.806088
Culicoides punctatus	58.61794	5.706579
Culicoides punctatus	58.61326	6.098003
Culicoides punctatus	58.55675	5.806088
Culicoides punctatus	58.61794	5.706579
Culicoides punctatus	58.79788	5.90309
Culicoides punctatus	58.55675	5.806088
Culicoides punctatus	63.58397	10.91978
Culicoides punctatus	58.61794	5.706579
Culicoides punctatus	59.20584	11.32002
Culicoides punctatus	58.79788	5.90309
Culicoides punctatus	58.61326	6.098003
Culicoides punctatus	58.61794	5.706579
Culicoides punctatus	58.55675	5.806088
Culicoides punctatus	58.61326	6.098003
Culicoides punctatus	59.18298	11.31991
Culicoides punctatus	58.55675	5.806088
Culicoides punctatus	58.79788	5.90309
Culicoides punctatus	58.55675	5.806088
Culicoides punctatus	58.79788	5.90309
Culicoides punctatus	58.55675	5.806088
Culicoides punctatus	59.51861	5.566979
Culicoides punctatus	58.55675	5.806088
Culicoides punctatus	60.12398	10.14998

Culicoides punctatus	58.61794	5.706579
Culicoides punctatus	58.61326	6.098003
Culicoides punctatus	58.55675	5.806088
Culicoides punctatus	58.61326	6.098003
Culicoides punctatus	58.61794	5.706579
Culicoides punctatus	56.4	9.29
Culicoides punctatus	54.23547	-2.87523
Culicoides punctatus	54.22272	-3.01147
Culicoides punctatus	56.43221	-2.861
Culicoides punctatus	56.35736	-3.21536
Culicoides punctatus	53.35903	-1.17506
Culicoides punctatus	53.44985	-1.32388
Culicoides punctatus	53.35997	-1.3253
Culicoides punctatus	53.44985	-1.32388
Culicoides punctatus	53.53609	-0.97087
Culicoides punctatus	58.3334	8.2399
Culicoides punctatus	58.1103	7.9342
Culicoides punctatus	58.0696	7.981
Culicoides punctatus	58.1103	7.9342
Culicoides punctatus	51.18446	4.63642
Culicoides punctatus	51.15884	4.69367
Culicoides punctatus	51.08564	4.95383
Culicoides punctatus	51.11861	4.66565
Culicoides punctatus	51.21351	4.1935
Culicoides punctatus	51.21351	4.1935
Culicoides punctatus	51.10875	3.75113
Culicoides punctatus	51.10875	3.75113
Culicoides punctatus	50.76562	3.1699
Culicoides punctatus	51.08564	4.95383
Culicoides punctatus	51.15884	4.69367
Culicoides punctatus	51.002	4.79206
Culicoides punctatus	51.002	4.79206
Culicoides punctatus	51.08564	4.95383
Culicoides punctatus	51.08564	4.95383
Culicoides punctatus	51.26369	4.36024
Culicoides punctatus	51.08564	4.95383
Culicoides punctatus	51.002	4.79206
Culicoides punctatus	51.002	4.79206
Culicoides punctatus	51.002	4.79206
Culicoides punctatus	51.002	4.79206
Culicoides punctatus	51.15884	4.69367
Culicoides punctatus	51.002	4.79206
Culicoides punctatus	51.002	4.79206
Culicoides punctatus	51.002	4.79206
Culicoides punctatus	51.002	4.79206
Culicoides punctatus	51.21351	4.1935
Culicoides punctatus	50.76562	3.1699

Culicoides punctatus	51.08564	4.95383
Culicoides punctatus	51.21351	4.1935
Culicoides punctatus	51.08564	4.95383
Culicoides punctatus	51.11861	4.66565
Culicoides punctatus	51.15185	4.86577
Culicoides punctatus	51.26369	4.36024
Culicoides punctatus	51.08564	4.95383
Culicoides punctatus	51.18446	4.63642
Culicoides punctatus	51.08564	4.95383
Culicoides punctatus	51.18446	4.63642
Culicoides punctatus	51.26369	4.36024
Culicoides punctatus	50.76562	3.1699
Culicoides punctatus	50.76562	3.1699
Culicoides punctatus	51.08564	4.95383
Culicoides punctatus	51.26369	4.36024
Culicoides punctatus	51.26369	4.36024
Culicoides punctatus	51.26369	4.36024
Culicoides punctatus	51.10875	3.75113
Culicoides punctatus	51.26369	4.36024
Culicoides punctatus	51.10875	3.75113
Culicoides punctatus	51.26369	4.36024
Culicoides punctatus	51.10875	3.75113
Culicoides punctatus	51.26369	4.36024
Culicoides punctatus	51.11861	4.66565
Culicoides punctatus	51.08564	4.95383
Culicoides punctatus	51.18446	4.63642
Culicoides punctatus	51.08564	4.95383
Culicoides punctatus	51.11861	4.66565
Culicoides punctatus	51.11861	4.66565
Culicoides punctatus	51.08564	4.95383
Culicoides punctatus	51.11861	4.66565
Culicoides punctatus	51.11861	4.66565
Culicoides punctatus	51.08564	4.95383
Culicoides punctatus	50.87615	4.37289
Culicoides punctatus	50.87615	4.37289
Culicoides punctatus	51.11861	4.66565
Culicoides punctatus	49.08	21.3
Culicoides punctatus	59.48	17.46
Culicoides punctatus	55.45	13.32
Culicoides punctatus	56.21	15.49
Culicoides punctatus	63.4	14.28
Culicoides punctatus	58.27	13.43
Culicoides punctatus	53.82977	-1.0215
Culicoides punctatus	54.27408	-2.13203
Culicoides punctatus	51.34629	3.295786
Culicoides punctatus	51.05637	3.723909
Culicoides punctatus	50.13583	4.745724

Culicoides punctatus	51.04022	3.81058
Culicoides punctatus	49.568	5.533
Culicoides punctatus	50.94541	3.744594
Culicoides punctatus	51.09429	2.589399
Culicoides punctatus	51.09468	2.589614
Culicoides punctatus	51.09408	2.586309
Culicoides punctatus	51.04022	3.81058
Culicoides punctatus	51.28829	5.189944
Culicoides punctatus	49.568	5.533
Culicoides punctatus	58.3124	22.39166
Culicoides punctatus	43.02077	41.01969
Culicoides punctatus	58.36838	26.70312
Culicoides punctatus	58.36838	26.70312
Culicoides punctatus	58.36838	26.70312
Culicoides punctatus	58.36838	26.70312
Culicoides punctatus	58.20403	26.104
Culicoides punctatus	57.90066	26.2816
Culicoides punctatus	58.33681	26.67591
Culicoides punctatus	58.36838	26.70312
Culicoides punctatus	58.83532	26.34079
Culicoides punctatus	58.39308	26.55607
Culicoides punctatus	57.90066	26.2816
Culicoides punctatus	59.27283	27.51737
Culicoides punctatus	53.1736	-3.42274
Culicoides punctatus	52.9538	-3.9864
Culicoides punctatus	52.50122	-3.65801
Culicoides punctatus	53.23301	-4.16414
Culicoides punctatus	53.09897	-3.78995
Culicoides punctatus	52.49342	-3.35281
Culicoides punctatus	53.09897	-3.78995
Culicoides punctatus	51.44476	-2.64529
Culicoides punctatus	51.55	-3.8042
Culicoides punctatus	52.18504	-3.39097
Culicoides punctatus	52.18504	-3.39097
Culicoides punctatus	52.18504	-3.39097
Culicoides punctatus	52.18504	-3.39097
Culicoides punctatus	52.18504	-3.39097
Culicoides punctatus	52.18504	-3.39097
Culicoides punctatus	52.18504	-3.39097
Culicoides punctatus	52.18504	-3.39097
Culicoides punctatus	52.33364	-2.26632
Culicoides punctatus	53.89375	-0.95039
Culicoides punctatus	52.33335	-2.25289
Culicoides punctatus	55.45	13.32
Culicoides punctatus	55.24	-2.18
Culicoides punctatus	52.03	1.08
Culicoides punctatus	51.73	0.43

Culicoides punctatus	55.24	-2.18
Culicoides punctatus	51.73	0.43
Culicoides punctatus	51.73	0.43
Culicoides punctatus	55.24	-2.18
Culicoides punctatus	55.24	-2.18
Culicoides punctatus	53.05	-1.77
Culicoides punctatus	55.24	-2.18
Culicoides punctatus	52.03	1.08
Culicoides punctatus	52.03	1.08
Culicoides punctatus	51.17	-3.94
Culicoides punctatus	55.24	-2.18
Culicoides punctatus	51.73	0.43
Culicoides punctatus	49.21	-2.04
Culicoides punctatus	55.24	-2.18
Culicoides punctatus	53.05	-1.77
Culicoides punctatus	51.73	0.43
Culicoides punctatus	52.03	1.08
Culicoides punctatus	51.73	0.43
Culicoides punctatus	52.03	1.08
Culicoides punctatus	55.24	-2.18
Culicoides punctatus	55.24	-2.18
Culicoides punctatus	55.24	-2.18
Culicoides punctatus	52.03	1.08
Culicoides punctatus	51.73	0.43
Culicoides punctatus	55.24	-2.18
Culicoides punctatus	54.33	-2.74
Culicoides punctatus	51.17	-3.94
Culicoides punctatus	50.64	-4.64
Culicoides punctatus	55.24	-2.18
Culicoides punctatus	54.63	-2.13
Culicoides punctatus	51.17	-3.94
Culicoides punctatus	54.33	-2.74
Culicoides punctatus	54.33	-2.74
Culicoides punctatus	54.33	-2.74
Culicoides punctatus	51.73	0.43
Culicoides punctatus	54.33	-2.74
Culicoides punctatus	54.33	-2.74
Culicoides punctatus	54.63	-2.13
Culicoides punctatus	51.17	-3.94
Culicoides punctatus	54.33	-2.74
Culicoides punctatus	51.22	0.87
Culicoides punctatus	51.73	0.43
Culicoides punctatus	55.24	-2.18
Culicoides punctatus	54.63	-2.13
Culicoides punctatus	54.33	-2.74
Culicoides punctatus	55.24	-2.18
Culicoides punctatus	51.17	-3.94

Culicoides punctatus	51.73	0.43
Culicoides punctatus	54.63	-2.13
Culicoides punctatus	51.22	0.87
Culicoides punctatus	54.33	-2.74
Culicoides punctatus	54.33	-2.74
Culicoides punctatus	51.17	-3.94
Culicoides punctatus	54.63	-2.13
Culicoides punctatus	54.33	-2.74
Culicoides punctatus	51.17	-3.94
Culicoides punctatus	54.63	-2.13
Culicoides punctatus	51.17	-3.94
Culicoides punctatus	54.63	-2.13
Culicoides punctatus	51.73	0.43
Culicoides punctatus	54.63	-2.13
Culicoides punctatus	51.17	-3.94
Culicoides punctatus	55.24	-2.18
Culicoides punctatus	55.24	-2.18
Culicoides punctatus	55.24	-2.18
Culicoides punctatus	54.63	-2.13
Culicoides punctatus	54.33	-2.74
Culicoides punctatus	54.33	-2.74
Culicoides punctatus	51.73	0.43
Culicoides punctatus	50.64	-4.64
Culicoides punctatus	54.33	-2.74
Culicoides punctatus	51.17	-3.94
Culicoides punctatus	51.73	0.43
Culicoides punctatus	51.73	0.43
Culicoides punctatus	54.63	-2.13
Culicoides punctatus	51.17	-3.94
Culicoides punctatus	55.24	-2.18
Culicoides punctatus	54.33	-2.74
Culicoides punctatus	51.73	0.43
Culicoides punctatus	55.24	-2.18
Culicoides punctatus	54.63	-2.13
Culicoides punctatus	54.63	-2.13
Culicoides punctatus	54.33	-2.74
Culicoides punctatus	51.22	0.87
Culicoides punctatus	50.64	-4.64
Culicoides punctatus	54.33	-2.74
Culicoides punctatus	50.64	-4.64
Culicoides punctatus	55.24	-2.18
Culicoides punctatus	54.63	-2.13
Culicoides punctatus	55.24	-2.18
Culicoides punctatus	51.73	0.43
Culicoides punctatus	55.24	-2.18
Culicoides punctatus	51.22	0.87
Culicoides punctatus	51.17	-3.94

Culicoides punctatus	50.64	-4.64
Culicoides punctatus	54.33	-2.74
Culicoides punctatus	54.63	-2.13
Culicoides punctatus	55.24	-2.18
Culicoides punctatus	55.24	-2.18
Culicoides punctatus	54.63	-2.13
Culicoides punctatus	51.17	-3.94
Culicoides punctatus	51.73	0.43
Culicoides punctatus	54.63	-2.13
Culicoides punctatus	51.22	0.87
Culicoides punctatus	55.24	-2.18
Culicoides punctatus	55.24	-2.18
Culicoides punctatus	55.24	-2.18
Culicoides punctatus	55.24	-2.18
Culicoides punctatus	55.24	-2.18
Culicoides punctatus	51.17	-3.94
Culicoides punctatus	54.63	-2.13
Culicoides punctatus	54.33	-2.74
Culicoides punctatus	54.33	-2.74
Culicoides punctatus	55.24	-2.18
Culicoides punctatus	51.73	0.43
Culicoides punctatus	54.33	-2.74
Culicoides punctatus	54.33	-2.74
Culicoides punctatus	55.24	-2.18
Culicoides punctatus	54.33	-2.74
Culicoides punctatus	51.73	0.43
Culicoides punctatus	51.17	-3.94
Culicoides punctatus	54.63	-2.13
Culicoides punctatus	54.33	-2.74
Culicoides punctatus	51.73	0.43
Culicoides punctatus	51.17	-3.94
Culicoides punctatus	51.17	-3.94
Culicoides punctatus	51.73	0.43
Culicoides punctatus	54.33	-2.74
Culicoides punctatus	54.63	-2.13
Culicoides punctatus	51.73	0.43
Culicoides punctatus	51.73	0.43
Culicoides punctatus	54.63	-2.13
Culicoides punctatus	51.17	-3.94
Culicoides punctatus	51.73	0.43
Culicoides punctatus	54.63	-2.13
Culicoides punctatus	51.17	-3.94
Culicoides punctatus	54.63	-2.13
Culicoides punctatus	51.73	0.43
Culicoides punctatus	54.33	-2.74
Culicoides punctatus	54.63	-2.13
Culicoides punctatus	54.63	-2.13

Culicoides punctatus	54.63	-2.13
Culicoides punctatus	54.33	-2.74
Culicoides punctatus	51.17	-3.94
Culicoides punctatus	51.22	0.87
Culicoides punctatus	54.33	-2.74
Culicoides punctatus	50.64	-4.64
Culicoides punctatus	51.22	0.87
Culicoides punctatus	55.24	-2.18
Culicoides punctatus	50.64	-4.64
Culicoides punctatus	54.33	-2.74
Culicoides punctatus	51.22	0.87
Culicoides punctatus	55.24	-2.18
Culicoides punctatus	51.17	-3.94
Culicoides punctatus	51.73	0.43
Culicoides punctatus	50.64	-4.64
Culicoides punctatus	51.17	-3.94
Culicoides punctatus	54.63	-2.13
Culicoides punctatus	55.24	-2.18
Culicoides punctatus	50.64	-4.64
Culicoides punctatus	51.73	0.43
Culicoides punctatus	51.22	0.87
Culicoides punctatus	55.24	-2.18
Culicoides punctatus	51.22	0.87
Culicoides punctatus	51.17	-3.94
Culicoides punctatus	54.63	-2.13
Culicoides punctatus	54.33	-2.74
Culicoides punctatus	54.33	-2.74
Culicoides punctatus	51.73	0.43
Culicoides punctatus	54.63	-2.13
Culicoides punctatus	51.73	0.43
Culicoides punctatus	51.22	0.87
Culicoides punctatus	54.63	-2.13
Culicoides punctatus	51.73	0.43
Culicoides punctatus	51.73	0.43
Culicoides punctatus	51.22	0.87
Culicoides punctatus	50.64	-4.64
Culicoides punctatus	51.17	-3.94
Culicoides punctatus	51.73	0.43
Culicoides punctatus	54.63	-2.13
Culicoides punctatus	51.73	0.43
Culicoides punctatus	54.63	-2.13
Culicoides punctatus	55.24	-2.18
Culicoides punctatus	50.64	-4.64
Culicoides punctatus	55.24	-2.18
Culicoides punctatus	51.73	0.43
Culicoides punctatus	51.73	0.43
Culicoides punctatus	51.73	0.43

Culicoides punctatus	54.63	-2.13
Culicoides punctatus	54.63	-2.13
Culicoides punctatus	55.24	-2.18
Culicoides punctatus	54.63	-2.13
Culicoides punctatus	50.64	-4.64
Culicoides punctatus	54.63	-2.13
Culicoides punctatus	51.22	0.87
Culicoides punctatus	55.24	-2.18
Culicoides punctatus	51.73	0.43
Culicoides punctatus	51.73	0.43
Culicoides punctatus	50.64	-4.64
Culicoides punctatus	55.24	-2.18
Culicoides punctatus	51.73	0.43
Culicoides punctatus	51.22	0.87
Culicoides punctatus	54.33	-2.74
Culicoides punctatus	50.64	-4.64
Culicoides punctatus	51.22	0.87
Culicoides punctatus	51.17	-3.94
Culicoides punctatus	51.73	0.43
Culicoides punctatus	54.33	-2.74
Culicoides punctatus	51.73	0.43
Culicoides punctatus	54.63	-2.13
Culicoides punctatus	51.73	0.43
Culicoides punctatus	51.22	0.87
Culicoides punctatus	50.64	-4.64
Culicoides punctatus	51.73	0.43
Culicoides punctatus	54.33	-2.74
Culicoides punctatus	51.73	0.43
Culicoides punctatus	51.17	-3.94
Culicoides punctatus	51.73	0.43
Culicoides punctatus	51.17	-3.94
Culicoides punctatus	51.73	0.43
Culicoides punctatus	54.63	-2.13
Culicoides punctatus	54.63	-2.13
Culicoides punctatus	54.33	-2.74
Culicoides punctatus	54.33	-2.74
Culicoides punctatus	50.64	-4.64
Culicoides punctatus	51.17	-3.94
Culicoides punctatus	51.73	0.43
Culicoides punctatus	51.17	-3.94
Culicoides punctatus	54.63	-2.13
Culicoides punctatus	55.24	-2.18
Culicoides punctatus	54.63	-2.13
Culicoides punctatus	51.73	0.43
Culicoides punctatus	51.17	-3.94
Culicoides punctatus	51.22	0.87
Culicoides punctatus	51.73	0.43

Culicoides punctatus	51.17	-3.94
Culicoides punctatus	51.73	0.43
Culicoides punctatus	51.17	-3.94
Culicoides punctatus	54.63	-2.13
Culicoides punctatus	51.22	0.87
Culicoides punctatus	51.73	0.43
Culicoides punctatus	51.17	-3.94
Culicoides punctatus	51.17	-3.94
Culicoides punctatus	51.73	0.43
Culicoides punctatus	51.22	0.87
Culicoides punctatus	55.24	-2.18
Culicoides punctatus	54.33	-2.74
Culicoides punctatus	51.73	0.43
Culicoides punctatus	51.73	0.43
Culicoides punctatus	51.22	0.87
Culicoides punctatus	51.73	0.43
Culicoides punctatus	51.17	-3.94
Culicoides punctatus	51.22	0.87
Culicoides punctatus	51.73	0.43
Culicoides punctatus	54.63	-2.13
Culicoides punctatus	55.24	-2.18
Culicoides punctatus	54.63	-2.13
Culicoides punctatus	54.63	-2.13
Culicoides punctatus	51.73	0.43
Culicoides punctatus	54.63	-2.13
Culicoides punctatus	54.63	-2.13
Culicoides punctatus	51.73	0.43
Culicoides punctatus	51.73	0.43
Culicoides punctatus	52.49	-3.44
Culicoides punctatus	51.17	-3.94
Culicoides punctatus	54.33	-2.74
Culicoides punctatus	50.64	-4.64
Culicoides punctatus	53.05	-1.77
Culicoides punctatus	51.17	-3.94
Culicoides punctatus	50.64	-4.64
Culicoides punctatus	51.17	-3.94
Culicoides punctatus	55.24	-2.18
Culicoides punctatus	54.63	-2.13
Culicoides punctatus	53.05	-1.77
Culicoides punctatus	55.24	-2.18
Culicoides punctatus	51.73	0.43
Culicoides punctatus	53.05	-1.77
Culicoides punctatus	54.33	-2.74
Culicoides punctatus	51.73	0.43
Culicoides punctatus	55.24	-2.18
Culicoides punctatus	51.73	0.43
Culicoides punctatus	51.73	0.43

Culicoides punctatus	51.17	-3.94
Culicoides punctatus	51.73	0.43
Culicoides punctatus	51.17	-3.94
Culicoides punctatus	55.24	-2.18
Culicoides punctatus	55.24	-2.18
Culicoides punctatus	54.33	-2.74
Culicoides punctatus	53.05	-1.77
Culicoides punctatus	50.64	-4.64
Culicoides punctatus	55.24	-2.18
Culicoides punctatus	52.73	-2.84
Culicoides punctatus	54.33	-2.74
Culicoides punctatus	52.03	1.08
Culicoides punctatus	55.24	-2.18
Culicoides punctatus	55.24	-2.18
Culicoides punctatus	51.17	-3.94
Culicoides punctatus	54.33	-2.74
Culicoides punctatus	53.05	-1.77
Culicoides punctatus	51.17	-3.94
Culicoides punctatus	52.39	-0.82
Culicoides punctatus	51.73	0.43
Culicoides punctatus	51.17	-3.94
Culicoides punctatus	55.24	-2.18
Culicoides punctatus	55.24	-2.18
Culicoides punctatus	54.33	-2.74
Culicoides punctatus	54.33	-2.74
Culicoides punctatus	51.17	-3.94
Culicoides punctatus	51.17	-3.94
Culicoides punctatus	51.17	-3.94
Culicoides punctatus	54.63	-2.13
Culicoides punctatus	52.03	1.08
Culicoides punctatus	55.24	-2.18
Culicoides punctatus	55.24	-2.18
Culicoides punctatus	52.03	1.08
Culicoides punctatus	52.03	1.08
Culicoides punctatus	51.17	-3.94
Culicoides punctatus	51.17	-3.94
Culicoides punctatus	50.64	-4.64
Culicoides punctatus	50.64	-4.64
Culicoides punctatus	53.05	-1.77
Culicoides punctatus	53.05	-1.77
Culicoides punctatus	50.64	-4.64
Culicoides punctatus	53.05	-1.77
Culicoides punctatus	54.63	-2.13
Culicoides punctatus	51.73	0.43
Culicoides punctatus	51.17	-3.94
Culicoides punctatus	52.49	-3.44
Culicoides punctatus	51.73	0.43

Culicoides punctatus	55.24	-2.18
Culicoides punctatus	53.05	-1.77
Culicoides punctatus	51.73	0.43
Culicoides punctatus	55.24	-2.18
Culicoides punctatus	53.05	-1.77
Culicoides punctatus	53.05	-1.77
Culicoides punctatus	51.73	0.43
Culicoides punctatus	54.33	-2.74
Culicoides punctatus	52.73	-2.84
Culicoides punctatus	55.24	-2.18
Culicoides punctatus	51.17	-3.94
Culicoides punctatus	51.73	0.43
Culicoides punctatus	51.73	0.43
Culicoides punctatus	54.33	-2.74
Culicoides punctatus	51.73	0.43
Culicoides punctatus	54.63	-2.13
Culicoides punctatus	51.17	-3.94
Culicoides punctatus	55.24	-2.18
Culicoides punctatus	55.24	-2.18
Culicoides punctatus	55.24	-2.18
Culicoides punctatus	53.05	-1.77
Culicoides punctatus	55.24	-2.18
Culicoides punctatus	54.33	-2.74
Culicoides punctatus	51.73	0.43
Culicoides punctatus	51.17	-3.94
Culicoides punctatus	55.24	-2.18
Culicoides punctatus	55.24	-2.18
Culicoides punctatus	51.73	0.43
Culicoides punctatus	51.22	0.87
Culicoides punctatus	51.73	0.43
Culicoides punctatus	51.17	-3.94
Culicoides punctatus	55.24	-2.18
Culicoides punctatus	51.73	0.43
Culicoides punctatus	50.64	-4.64
Culicoides punctatus	55.24	-2.18
Culicoides punctatus	54.63	-2.13
Culicoides punctatus	55.24	-2.18
Culicoides punctatus	55.24	-2.18
Culicoides punctatus	51.22	0.87
Culicoides punctatus	55.24	-2.18
Culicoides punctatus	54.33	-2.74
Culicoides punctatus	52.03	1.08
Culicoides punctatus	54.33	-2.74
Culicoides punctatus	52.03	1.08
Culicoides punctatus	51.17	-3.94
Culicoides punctatus	55.24	-2.18
Culicoides punctatus	51.17	-3.94

Culicoides punctatus	54.33	-2.74
Culicoides punctatus	53.05	-1.77
Culicoides punctatus	50.64	-4.64
Culicoides punctatus	55.24	-2.18
Culicoides punctatus	52.49	-3.44
Culicoides punctatus	51.73	0.43
Culicoides punctatus	55.24	-2.18
Culicoides punctatus	55.24	-2.18
Culicoides punctatus	50.64	-4.64
Culicoides punctatus	55.24	-2.18
Culicoides punctatus	54.63	-2.13
Culicoides punctatus	51.17	-3.94
Culicoides punctatus	51.73	0.43
Culicoides punctatus	55.24	-2.18
Culicoides punctatus	51.17	-3.94
Culicoides punctatus	55.24	-2.18
Culicoides punctatus	54.33	-2.74
Culicoides punctatus	54.63	-2.13
Culicoides punctatus	49.21	-2.04
Culicoides punctatus	54.33	-2.74
Culicoides punctatus	51.17	-3.94
Culicoides punctatus	49.21	-2.04
Culicoides punctatus	55.24	-2.18
Culicoides punctatus	51.17	-3.94
Culicoides punctatus	51.17	-3.94
Culicoides punctatus	51.17	-3.94
Culicoides punctatus	51.22	0.87
Culicoides punctatus	51.17	-3.94
Culicoides punctatus	54.33	-2.74
Culicoides punctatus	51.17	-3.94
Culicoides punctatus	51.73	0.43
Culicoides punctatus	51.17	-3.94
Culicoides punctatus	49.22	-2.22
Culicoides punctatus	54.63	-2.13
Culicoides punctatus	54.63	-2.13
Culicoides punctatus	54.63	-2.13
Culicoides punctatus	49.21	-2.04
Culicoides punctatus	50.64	-4.64
Culicoides punctatus	50.64	-4.64
Culicoides punctatus	51.73	0.43
Culicoides punctatus	49.22	-2.14
Culicoides punctatus	54.33	-2.74
Culicoides punctatus	55.24	-2.18
Culicoides punctatus	54.33	-2.74
Culicoides punctatus	55.24	-2.18
Culicoides punctatus	55.24	-2.18
Culicoides punctatus	51.17	-3.94

Culicoides punctatus	51.17	-3.94
Culicoides punctatus	54.63	-2.13
Culicoides punctatus	54.63	-2.13
Culicoides punctatus	54.33	-2.74
Culicoides punctatus	55.24	-2.18
Culicoides punctatus	51.73	0.43
Culicoides punctatus	49.22	-2.14
Culicoides punctatus	55.24	-2.18
Culicoides punctatus	49.22	-2.14
Culicoides punctatus	55.24	-2.18
Culicoides punctatus	51.17	-3.94
Culicoides punctatus	51.17	-3.94
Culicoides punctatus	54.33	-2.74
Culicoides punctatus	54.63	-2.13
Culicoides punctatus	55.24	-2.18
Culicoides punctatus	49.22	-2.22
Culicoides punctatus	55.24	-2.18
Culicoides punctatus	54.63	-2.13
Culicoides punctatus	51.22	0.87
Culicoides punctatus	51.17	-3.94
Culicoides punctatus	49.22	-2.22
Culicoides punctatus	49.22	-2.14
Culicoides punctatus	51.73	0.43
Culicoides punctatus	54.63	-2.13
Culicoides punctatus	51.22	0.87
Culicoides punctatus	55.24	-2.18
Culicoides punctatus	51.17	-3.94
Culicoides punctatus	54.63	-2.13
Culicoides punctatus	49.22	-2.14
Culicoides punctatus	51.17	-3.94
Culicoides punctatus	51.17	-3.94
Culicoides punctatus	55.24	-2.18
Culicoides punctatus	51.17	-3.94
Culicoides punctatus	54.63	-2.13
Culicoides punctatus	49.22	-2.14
Culicoides punctatus	55.24	-2.18
Culicoides punctatus	55.24	-2.18
Culicoides punctatus	51.17	-3.94
Culicoides punctatus	54.33	-2.74
Culicoides punctatus	50.64	-4.64
Culicoides punctatus	54.33	-2.74
Culicoides punctatus	49.21	-2.04
Culicoides punctatus	55.24	-2.18
Culicoides punctatus	54.63	-2.13
Culicoides punctatus	51.17	-3.94
Culicoides punctatus	55.24	-2.18
Culicoides punctatus	51.17	-3.94

Culicoides punctatus	54.33	-2.74
Culicoides punctatus	55.24	-2.18
Culicoides punctatus	54.63	-2.13
Culicoides punctatus	50.64	-4.64
Culicoides punctatus	51.73	0.43
Culicoides punctatus	49.22	-2.22
Culicoides punctatus	51.17	-3.94
Culicoides punctatus	51.73	0.43
Culicoides punctatus	51.22	0.87
Culicoides punctatus	54.63	-2.13
Culicoides punctatus	54.33	-2.74
Culicoides punctatus	51.17	-3.94
Culicoides punctatus	50.64	-4.64
Culicoides punctatus	55.24	-2.18
Culicoides punctatus	50.64	-4.64
Culicoides punctatus	54.33	-2.74
Culicoides punctatus	51.22	0.87
Culicoides punctatus	54.33	-2.74
Culicoides punctatus	51.17	-3.94
Culicoides punctatus	51.22	0.87
Culicoides punctatus	50.64	-4.64
Culicoides punctatus	55.24	-2.18
Culicoides punctatus	55.24	-2.18
Culicoides punctatus	51.17	-3.94
Culicoides punctatus	51.73	0.43
Culicoides punctatus	49.22	-2.14
Culicoides punctatus	49.22	-2.14
Culicoides punctatus	54.33	-2.74
Culicoides punctatus	51.22	0.87
Culicoides punctatus	54.63	-2.13
Culicoides punctatus	51.17	-3.94
Culicoides punctatus	51.22	0.87
Culicoides punctatus	54.63	-2.13
Culicoides punctatus	51.22	0.87
Culicoides punctatus	54.33	-2.74
Culicoides punctatus	54.63	-2.13
Culicoides punctatus	50.64	-4.64
Culicoides punctatus	54.33	-2.74
Culicoides punctatus	49.22	-2.14
Culicoides punctatus	49.22	-2.14
Culicoides punctatus	49.21	-2.04
Culicoides punctatus	49.21	-2.04
Culicoides punctatus	54.33	-2.74
Culicoides punctatus	49.22	-2.14
Culicoides punctatus	54.33	-2.74
Culicoides punctatus	49.22	-2.14
Culicoides punctatus	55.24	-2.18

Culicoides punctatus	49.21	-2.04
Culicoides punctatus	51.22	0.87
Culicoides punctatus	49.22	-2.22
Culicoides punctatus	49.22	-2.14
Culicoides punctatus	51.73	0.43
Culicoides punctatus	55.24	-2.18
Culicoides punctatus	54.63	-2.13
Culicoides punctatus	54.63	-2.13
Culicoides punctatus	55.24	-2.18
Culicoides punctatus	51.17	-3.94
Culicoides punctatus	55.24	-2.18
Culicoides punctatus	51.17	-3.94
Culicoides punctatus	49.22	-2.14
Culicoides punctatus	54.33	-2.74
Culicoides punctatus	50.64	-4.64
Culicoides punctatus	54.33	-2.74
Culicoides punctatus	51.73	0.43
Culicoides punctatus	49.22	-2.22
Culicoides punctatus	54.63	-2.13
Culicoides punctatus	55.24	-2.18
Culicoides punctatus	49.22	-2.22
Culicoides punctatus	51.73	0.43
Culicoides punctatus	54.63	-2.13
Culicoides punctatus	51.73	0.43
Culicoides punctatus	54.33	-2.74
Culicoides punctatus	49.21	-2.04
Culicoides punctatus	54.63	-2.13
Culicoides punctatus	50.64	-4.64
Culicoides punctatus	49.22	-2.14
Culicoides punctatus	54.63	-2.13
Culicoides punctatus	49.22	-2.14
Culicoides punctatus	49.22	-2.22
Culicoides punctatus	55.24	-2.18
Culicoides punctatus	54.63	-2.13
Culicoides punctatus	49.21	-2.04
Culicoides punctatus	51.73	0.43
Culicoides punctatus	49.22	-2.22
Culicoides punctatus	51.73	0.43
Culicoides punctatus	49.21	-2.04
Culicoides punctatus	51.17	-3.94
Culicoides punctatus	51.22	0.87
Culicoides punctatus	49.21	-2.04
Culicoides punctatus	49.22	-2.14
Culicoides punctatus	50.64	-4.64
Culicoides punctatus	51.73	0.43
Culicoides punctatus	49.22	-2.14
Culicoides punctatus	51.22	0.87

Culicoides punctatus	51.73	0.43
Culicoides punctatus	54.63	-2.13
Culicoides punctatus	54.63	-2.13
Culicoides punctatus	54.33	-2.74
Culicoides punctatus	51.73	0.43
Culicoides punctatus	49.22	-2.14
Culicoides punctatus	51.73	0.43
Culicoides punctatus	54.33	-2.74
Culicoides punctatus	49.21	-2.04
Culicoides punctatus	50.64	-4.64
Culicoides punctatus	49.22	-2.22
Culicoides punctatus	51.17	-3.94
Culicoides punctatus	54.33	-2.74
Culicoides punctatus	51.73	0.43
Culicoides punctatus	49.21	-2.04
Culicoides punctatus	54.33	-2.74
Culicoides punctatus	51.73	0.43
Culicoides punctatus	49.22	-2.22
Culicoides punctatus	51.17	-3.94
Culicoides punctatus	54.33	-2.74
Culicoides punctatus	54.33	-2.74
Culicoides punctatus	54.33	-2.74
Culicoides punctatus	54.63	-2.13
Culicoides punctatus	54.63	-2.13
Culicoides punctatus	54.63	-2.13
Culicoides punctatus	55.24	-2.18
Culicoides punctatus	50.64	-4.64
Culicoides punctatus	54.33	-2.74
Culicoides punctatus	51.17	-3.94
Culicoides punctatus	51.73	0.43
Culicoides punctatus	49.22	-2.14
Culicoides punctatus	51.73	0.43
Culicoides punctatus	51.17	-3.94
Culicoides punctatus	50.64	-4.64
Culicoides punctatus	49.22	-2.14
Culicoides punctatus	51.17	-3.94
Culicoides punctatus	51.73	0.43
Culicoides punctatus	55.24	-2.18
Culicoides punctatus	50.64	-4.64
Culicoides punctatus	49.22	-2.14
Culicoides punctatus	49.21	-2.04
Culicoides punctatus	51.17	-3.94
Culicoides punctatus	50.64	-4.64
Culicoides punctatus	51.73	0.43
Culicoides punctatus	50.64	-4.64
Culicoides punctatus	49.21	-2.04
Culicoides punctatus	49.22	-2.22

Culicoides punctatus	51.17	-3.94
Culicoides punctatus	51.17	-3.94
Culicoides punctatus	51.22	0.87
Culicoides punctatus	55.24	-2.18
Culicoides punctatus	51.73	0.43
Culicoides punctatus	54.33	-2.74
Culicoides punctatus	54.63	-2.13
Culicoides punctatus	51.73	0.43
Culicoides punctatus	49.22	-2.22
Culicoides punctatus	49.21	-2.04
Culicoides punctatus	54.33	-2.74
Culicoides punctatus	51.17	-3.94
Culicoides punctatus	51.22	0.87
Culicoides punctatus	54.63	-2.13
Culicoides punctatus	55.24	-2.18
Culicoides punctatus	55.24	-2.18
Culicoides punctatus	50.64	-4.64
Culicoides punctatus	50.64	-4.64
Culicoides punctatus	51.17	-3.94
Culicoides punctatus	54.33	-2.74
Culicoides punctatus	50.64	-4.64
Culicoides punctatus	49.21	-2.04
Culicoides punctatus	49.22	-2.22
Culicoides punctatus	55.24	-2.18
Culicoides punctatus	54.33	-2.74
Culicoides punctatus	49.22	-2.14
Culicoides punctatus	49.22	-2.14
Culicoides punctatus	51.22	0.87
Culicoides punctatus	51.73	0.43
Culicoides punctatus	54.63	-2.13
Culicoides punctatus	51.17	-3.94
Culicoides punctatus	49.21	-2.04
Culicoides punctatus	51.22	0.87
Culicoides punctatus	51.73	0.43
Culicoides punctatus	54.33	-2.74
Culicoides punctatus	51.17	-3.94
Culicoides punctatus	51.22	0.87
Culicoides punctatus	54.63	-2.13
Culicoides punctatus	49.22	-2.14
Culicoides punctatus	51.17	-3.94
Culicoides punctatus	50.64	-4.64
Culicoides punctatus	51.17	-3.94
Culicoides punctatus	50.64	-4.64
Culicoides punctatus	51.73	0.43
Culicoides punctatus	51.73	0.43
Culicoides punctatus	55.24	-2.18
Culicoides punctatus	51.22	0.87

Culicoides punctatus	54.33	-2.74
Culicoides punctatus	51.17	-3.94
Culicoides punctatus	51.73	0.43
Culicoides punctatus	54.33	-2.74
Culicoides punctatus	54.33	-2.74
Culicoides punctatus	55.24	-2.18
Culicoides punctatus	52.39	-0.82
Culicoides punctatus	52.49	-3.44
Culicoides punctatus	52.49	-3.44
Culicoides punctatus	54.63	-2.13
Culicoides punctatus	55.24	-2.18
Culicoides punctatus	52.73	-2.84
Culicoides punctatus	52.49	-3.44
Culicoides punctatus	52.73	-2.84
Culicoides punctatus	54.33	-2.74
Culicoides punctatus	54.33	-2.74
Culicoides punctatus	54.63	-2.13
Culicoides punctatus	54.33	-2.74
Culicoides punctatus	51.17	-3.94
Culicoides punctatus	55.24	-2.18
Culicoides punctatus	54.33	-2.74
Culicoides punctatus	52.49	-3.44
Culicoides punctatus	55.24	-2.18
Culicoides punctatus	54.33	-2.74
Culicoides punctatus	51.17	-3.94
Culicoides punctatus	55.24	-2.18
Culicoides punctatus	51.73	0.43
Culicoides punctatus	51.17	-3.94
Culicoides punctatus	52.39	-0.82
Culicoides punctatus	54.33	-2.74
Culicoides punctatus	54.33	-2.74
Culicoides punctatus	51.17	-3.94
Culicoides punctatus	52.49	-3.44
Culicoides punctatus	55.24	-2.18
Culicoides punctatus	54.63	-2.13
Culicoides punctatus	54.63	-2.13
Culicoides punctatus	51.22	0.87
Culicoides punctatus	51.17	-3.94
Culicoides punctatus	52.49	-3.44
Culicoides punctatus	51.22	0.87
Culicoides punctatus	50.64	-4.64
Culicoides punctatus	52.49	-3.44
Culicoides punctatus	55.24	-2.18
Culicoides punctatus	51.17	-3.94
Culicoides punctatus	54.63	-2.13
Culicoides punctatus	51.22	0.87
Culicoides punctatus	54.33	-2.74

Culicoides punctatus	50.64	-4.64
Culicoides punctatus	52.49	-3.44
Culicoides punctatus	54.33	-2.74
Culicoides punctatus	55.24	-2.18
Culicoides punctatus	52.49	-3.44
Culicoides punctatus	54.63	-2.13
Culicoides punctatus	54.33	-2.74
Culicoides punctatus	54.63	-2.13
Culicoides punctatus	55.24	-2.18
Culicoides punctatus	54.63	-2.13
Culicoides punctatus	55.24	-2.18
Culicoides punctatus	52.49	-3.44
Culicoides punctatus	55.24	-2.18
Culicoides punctatus	55.24	-2.18
Culicoides punctatus	54.33	-2.74
Culicoides punctatus	55.24	-2.18
Culicoides punctatus	51.17	-3.94
Culicoides punctatus	52.49	-3.44
Culicoides punctatus	54.63	-2.13
Culicoides punctatus	55.24	-2.18
Culicoides punctatus	52.73	-2.84
Culicoides punctatus	54.63	-2.13
Culicoides punctatus	52.73	-2.84
Culicoides punctatus	51.17	-3.94
Culicoides punctatus	52.49	-3.44
Culicoides punctatus	52.26	0.56
Culicoides punctatus	55.24	-2.18
Culicoides punctatus	52.73	-2.84
Culicoides punctatus	52.49	-3.44
Culicoides punctatus	54.33	-2.74
Culicoides punctatus	52.49	-3.44
Culicoides punctatus	54.33	-2.74
Culicoides punctatus	52.49	-3.44
Culicoides punctatus	55.24	-2.18
Culicoides punctatus	52.49	-3.44
Culicoides punctatus	52.49	-3.44
Culicoides punctatus	54.63	-2.13
Culicoides punctatus	55.24	-2.18
Culicoides punctatus	54.33	-2.74
Culicoides punctatus	51.17	-3.94
Culicoides punctatus	54.63	-2.13
Culicoides punctatus	51.73	0.43
Culicoides punctatus	54.33	-2.74
Culicoides punctatus	54.33	-2.74
Culicoides punctatus	54.63	-2.13
Culicoides punctatus	52.73	-2.84
Culicoides punctatus	54.33	-2.74

Culicoides punctatus	54.33	-2.74
Culicoides punctatus	54.33	-2.74
Culicoides punctatus	55.24	-2.18
Culicoides punctatus	52.49	-3.44
Culicoides punctatus	52.73	-2.84
Culicoides punctatus	52.49	-3.44
Culicoides punctatus	54.63	-2.13
Culicoides punctatus	54.33	-2.74
Culicoides punctatus	54.33	-2.74
Culicoides punctatus	54.63	-2.13
Culicoides punctatus	54.33	-2.74
Culicoides punctatus	55.24	-2.18
Culicoides punctatus	52.26	0.56
Culicoides punctatus	52.49	-3.44
Culicoides punctatus	54.63	-2.13
Culicoides punctatus	54.63	-2.13
Culicoides punctatus	54.63	-2.13
Culicoides punctatus	52.39	-0.82
Culicoides punctatus	55.24	-2.18
Culicoides punctatus	52.49	-3.44
Culicoides punctatus	55.24	-2.18
Culicoides punctatus	52.49	-3.44
Culicoides punctatus	52.49	-3.44
Culicoides punctatus	52.49	-3.44
Culicoides punctatus	54.33	-2.74
Culicoides punctatus	51.45	4.05
Culicoides punctatus	61.4242	11.1011
Culicoides punctatus	32.47139	3.699444
Culicoides punctatus	34.4375	1.554722
Culicoides punctatus	33.49556	0.269444
Culicoides punctatus	34.86472	5.788611
Culicoides punctatus	30.02083	31.29389
Culicoides punctatus	30.10083	31.45667
Culicoides punctatus	31.11278	29.8425
Culicoides punctatus	28.38528	28.91028
Culicoides punctatus	23.68722	25.94056
Culicoides punctatus	23.564	26.1559
Culicoides punctatus	28.47	34.44
Culicoides punctatus	28.52	34.5
Culicoides punctatus	30.07417	31.2425
Culicoides punctatus	29.87833	31.31361
Culicoides punctatus	30.07417	31.2425
Culicoides punctatus	29.95972	31.33056
Culicoides punctatus	22.18333	36.35
Culicoides punctatus	22.18333	36.35
Culicoides punctatus	30.92361	29.54222
Culicoides punctatus	29.95972	31.33056

Culicoides punctatus	31.15917	29.93056
Culicoides punctatus	29.97722	31.13222
Culicoides punctatus	22.33972	31.55583
Culicoides punctatus	29.95972	31.33056
Culicoides punctatus	28.38528	28.91028
Culicoides punctatus	28.57278	33.93
Culicoides punctatus	28.58167	33.92583
Culicoides punctatus	28.56944	33.9275
Culicoides punctatus	29.95972	31.33056
Culicoides punctatus	25.455	30.4925
Culicoides punctatus	28.38528	28.90972
Culicoides punctatus	30.20111	31.35528
Culicoides punctatus	25.84389	30.78028
Culicoides punctatus	23.14833	35.38528
Culicoides punctatus	30.07083	31.245
Culicoides punctatus	30.07056	31.24417
Culicoides punctatus	29.95972	31.33056
Culicoides punctatus	26.67972	33.10139
Culicoides punctatus	31.15917	29.93056
Culicoides punctatus	29.87167	31.38472
Culicoides punctatus	27.65111	31.00528
Culicoides punctatus	25.51667	29.16667
Culicoides punctatus	26.67972	33.10139
Culicoides punctatus	25.51	29.14139
Culicoides punctatus	31.0425	29.80194
Culicoides punctatus	30.02194	32.45444
Culicoides punctatus	30.02722	31.28167
Culicoides punctatus	27.28306	33.52861
Culicoides punctatus	30.03389	32.45389
Culicoides punctatus	30.03083	32.44694
Culicoides punctatus	25.45639	30.58222
Culicoides punctatus	30.02722	31.28167
Culicoides punctatus	25.70722	32.46333
Culicoides punctatus	29.06778	32.89472
Culicoides punctatus	30.92361	29.54222
Culicoides punctatus	30.01444	32.44556
Culicoides punctatus	30.04111	32.40139
Culicoides punctatus	29.87167	31.38472
Culicoides punctatus	29.95972	31.33056
Culicoides punctatus	30.01861	31.30417
Culicoides punctatus	29.87889	31.31333
Culicoides punctatus	30.0025	31.28
Culicoides punctatus	25.45389	30.58278
Culicoides punctatus	28.19111	30.29444
Culicoides punctatus	30.00278	32.41167
Culicoides punctatus	25.455	30.4925
Culicoides punctatus	30.02056	31.3

Culicoides punctatus	29.97694	31.12972
Culicoides punctatus	29.86806	31.34556
Culicoides punctatus	26.10417	34.25972
Culicoides punctatus	25.66444	32.77333
Culicoides punctatus	26.03917	34.30389
Culicoides punctatus	30.02944	32.48694
Culicoides punctatus	25.71194	32.57389
Culicoides punctatus	29.96083	32.43472
Culicoides punctatus	30.00028	31.28083
Culicoides punctatus	30.0225	31.30528
Culicoides punctatus	30.12722	31.47306
Culicoides punctatus	25.03778	34.81278
Culicoides punctatus	31.575	25.15917
Culicoides punctatus	28.39361	30.53
Culicoides punctatus	29.24056	25.55194
Culicoides punctatus	30.01694	31.30361
Culicoides punctatus	28.32194	28.95806
Culicoides punctatus	29.96083	32.43472
Culicoides punctatus	25.73028	32.58194
Culicoides punctatus	26.09611	34.26361
Culicoides punctatus	25.64167	32.72611
Culicoides punctatus	22.33972	31.55583
Culicoides punctatus	22.75639	31.78861
Culicoides punctatus	30.045	32.44861
Culicoides punctatus	30.07278	31.5525
Culicoides punctatus	30.05528	32.375
Culicoides punctatus	30.05	32.45222
Culicoides punctatus	28.10972	30.50028
Culicoides punctatus	28.48	30.42917
Culicoides punctatus	27.95167	34.27278
Culicoides punctatus	33.13333	47.36667
Culicoides punctatus	32.63028	52.07972
Culicoides punctatus	28.65278	57.83972
Culicoides punctatus	32.81111	59.47
Culicoides punctatus	33.96556	51.32556
Culicoides punctatus	27.21806	60.66111
Culicoides punctatus	27.35056	62.35889
Culicoides punctatus	34.40056	60.24083
Culicoides punctatus	28.60806	61.00028
Culicoides punctatus	38.9075	46.865
Culicoides punctatus	37.36861	54.95722
Culicoides punctatus	34.53806	43.48306
Culicoides punctatus	33.41611	35.85694
Culicoides punctatus	30.82944	35.03861
Culicoides punctatus	30.99806	35.18944
Culicoides punctatus	33.01667	35.35
Culicoides punctatus	30.87306	34.43333

Culicoides punctatus	31.65417	35.37222
Culicoides punctatus	31.34972	53.33972
Culicoides punctatus	30.71389	34.87556
Culicoides punctatus	31.91667	36.83333
Culicoides punctatus	30.68333	35.65
Culicoides punctatus	30.66667	35.6
Culicoides punctatus	30.4	35.5
Culicoides punctatus	31.31667	35.75
Culicoides punctatus	30.5	35.51667
Culicoides punctatus	30.5	35.51667
Culicoides punctatus	30.83333	35.6
Culicoides punctatus	30.68333	35.65
Culicoides punctatus	30.68333	35.65
Culicoides punctatus	30.51667	35.55
Culicoides punctatus	30.51667	35.55
Culicoides punctatus	29.61667	35.96667
Culicoides punctatus	31.91667	36.83333
Culicoides punctatus	31.91667	36.83333
Culicoides punctatus	31.91667	36.83333
Culicoides punctatus	30.73333	35.56667
Culicoides punctatus	30.73333	35.56667
Culicoides punctatus	30.4	35.5
Culicoides punctatus	31.81667	36.1
Culicoides punctatus	31.81667	36.1
Culicoides punctatus	31.81667	36.1
Culicoides punctatus	31.81667	36.1
Culicoides punctatus	31.25	36.05
Culicoides punctatus	32.01667	36.05
Culicoides punctatus	32.01667	36.05
Culicoides punctatus	30.5	35.51667
Culicoides punctatus	30.5	35.51667
Culicoides punctatus	31.36667	36.11667
Culicoides punctatus	30.83333	35.6
Culicoides punctatus	32.03333	36.06667
Culicoides punctatus	32.03333	36.06667
Culicoides punctatus	30.68333	35.65
Culicoides punctatus	32.03333	35.88333
Culicoides punctatus	31.1	35.7
Culicoides punctatus	30.51667	35.55
Culicoides punctatus	29.61667	35.96667
Culicoides punctatus	29.48333	35.61667
Culicoides punctatus	29.48333	35.61667
Culicoides punctatus	32.01667	35.78333
Culicoides punctatus	30.4	35.5
Culicoides punctatus	31.95	35.93333
Culicoides punctatus	31.95	35.93333
Culicoides punctatus	30.5	35.51667

Culicoides punctatus	32.06667	35.56667
Culicoides punctatus	32.06667	35.56667
Culicoides punctatus	30.51667	35.55
Culicoides punctatus	32.45	36.23333
Culicoides punctatus	32.33333	36.36667
Culicoides punctatus	32.41667	36.5
Culicoides punctatus	31.25	36.05
Culicoides punctatus	31.60694	36.35389
Culicoides punctatus	30.15944	35.71028
Culicoides punctatus	29.05583	47.85417
Culicoides punctatus	29.65	47.52
Culicoides punctatus	29.19583	47.555
Culicoides punctatus	28.65333	48.05056
Culicoides punctatus	29.21944	47.54917
Culicoides punctatus	34.36333	36.41444
Culicoides punctatus	33.90306	35.50083
Culicoides punctatus	23.21389	21.79889
Culicoides punctatus	32.40639	13.40917
Culicoides punctatus	33.90611	35.69972
Culicoides punctatus	30.06833	5.6525
Culicoides punctatus	33.90056	2.543056
Culicoides punctatus	28.49306	11.10472
Culicoides punctatus	28.43361	9.2925
Culicoides punctatus	22.81917	14.38806
Culicoides punctatus	23.545	58.33667
Culicoides punctatus	21.44222	58.555
Culicoides punctatus	24.26972	56.58083
Culicoides punctatus	17.02417	54.22583
Culicoides punctatus	23.08667	58.10556
Culicoides punctatus	24.26972	56.58083
Culicoides punctatus	17.90444	54.34083
Culicoides punctatus	21.03111	58.22389
Culicoides punctatus	23.48806	58.27083
Culicoides punctatus	23.58389	58.52139
Culicoides punctatus	23.58333	58.46667
Culicoides punctatus	31.79806	35.27389
Culicoides punctatus	31.73639	35.29444
Culicoides punctatus	24.89972	51.17972
Culicoides punctatus	25.95139	51.40583
Culicoides punctatus	24.89972	51.17972
Culicoides punctatus	25.35917	51.21333
Culicoides punctatus	20.91139	43.14472
Culicoides punctatus	21.33778	39.28972
Culicoides punctatus	24.17389	50.99972
Culicoides punctatus	18.84667	42.80917
Culicoides punctatus	22.32194	50.67556
Culicoides punctatus	20.02833	41.47222

Culicoides punctatus	19.75583	41.42639
Culicoides punctatus	20.025	41.43444
Culicoides punctatus	20.02861	41.47028
Culicoides punctatus	23.45	46.68333
Culicoides punctatus	23.7	46.4
Culicoides punctatus	21.08639	40.29167
Culicoides punctatus	21.17583	40.39
Culicoides punctatus	20.7	49.96667
Culicoides punctatus	20.02861	41.47028
Culicoides punctatus	20.02861	41.47028
Culicoides punctatus	24.73944	46.54
Culicoides punctatus	23.52139	46.85194
Culicoides punctatus	23.52139	46.85194
Culicoides punctatus	21.46278	39.76333
Culicoides punctatus	20.03667	41.47333
Culicoides punctatus	24.68611	46.51611
Culicoides punctatus	28.595	34.83194
Culicoides punctatus	28.74194	34.84222
Culicoides punctatus	12.10833	24.98556
Culicoides punctatus	13.08528	30.34861
Culicoides punctatus	15.52417	32.71083
Culicoides punctatus	13.08361	30.35
Culicoides punctatus	34.55972	38.26667
Culicoides punctatus	36.13972	37.22
Culicoides punctatus	36.45833	36.29056
Culicoides punctatus	36.98889	38.82528
Culicoides punctatus	36.40222	36.34972
Culicoides punctatus	36.89472	30.70972
Culicoides punctatus	36.89472	30.70972
Culicoides punctatus	36.20028	36.17639
Culicoides punctatus	36.75	36.33333
Culicoides punctatus	15.36222	44.14028
Culicoides punctatus	24.98694	56.32417
Culicoides punctatus	25.41611	56.35889
Culicoides punctatus	25.39056	56.30972
Culicoides punctatus	25.38944	56.31056
Culicoides punctatus	25.38889	56.31056
Culicoides punctatus	25.38917	56.31083
Culicoides punctatus	24.11	55.76
Culicoides punctatus	24.05694	55.77778
Culicoides punctatus	24.08111	55.80667
Culicoides punctatus	24.55639	46.4825
